# Mental disorders in judicial workers: analysis of sickness absence in a cohort study

**DOI:** 10.11606/S01518-8787.2023057004737err

**Published:** 2023-11-17

In the article **“Mental disorders in judicial workers: analysis of sickness absence in a cohort study”**, https://doi.org/10.11606/s1518-8787.2023057004737, published on the Revista de Saúde Pública.2023;57:72, the RSP corrects on page 1 the authorship order and how to cite, and on page 10 the authors’ contributions.

**On page 1**

**Where it reads:**

Bruna Ferreira Melo^I^, Kionna Oliveira Bernardes Santos^I^, Rita de Cássia Pereira Fernandes^I^, Verônica Maria Cadena de Lima^I^, Susan Stock^II^

**It should read:**

Bruna Ferreira Melo^I^, Kionna Oliveira Bernardes Santos^I^, Susan Stock^II^, Verônica Maria Cadena de Lima^I^, Rita de Cássia Pereira Fernandes^I^

**Where it reads:**

**How to cite:** Melo BF, Santos KOB, Fernandes RCP, Lima VMC, Stock S. Mental disorders in judicial workers: analysis of sickness absence in a cohort study. Rev Saude Publica. 2023;57:72. https://doi.org/10.11606/s15188787.2023057004737

**It should read:**

**How to cite:** Melo BF, Santos KOB, Stock S, Lima VMC, Fernandes RCP. Mental disorders in judicial workers: analysis of sickness absence in a cohort study. Rev Saude Publica. 2023;57:72. https://doi.org/10.11606/s15188787.2023057004737

**On page 10**

**Where it reads:**

**Authors’ Contribution:** Study design and planning: KOB; RCP. Data collection, analysis, and interpretation: BFM; VMCL; KOB. Manuscript drafting or review: BFM; KOB; RCP. Approval of the final version: KOB; RCP; SS. Public responsibility for the content of the article: BFM; KOB.

**It should read:**

**Authors’ Contribution:** Study design and planning: KOB; RCP. Data collection, analysis and interpretation: BFM; VMCL; KOB; RCP; SS. Manuscript drafting or review: BFM; KOB; RCP; SS. Approval of the final version: BFM; KOB; RCP; SS. Public responsibility for the content of the article: BFM; KOB.

